# Medical Image Classification Based on Deep Features Extracted by Deep Model and Statistic Feature Fusion with Multilayer Perceptron^‬^

**DOI:** 10.1155/2018/2061516

**Published:** 2018-09-12

**Authors:** ZhiFei Lai, HuiFang Deng

**Affiliations:** Department of Computer Science and Engineering, South China University of Technology, Guangzhou 510006, China

## Abstract

Medical image classification is a key technique of Computer-Aided Diagnosis (CAD) systems. Traditional methods rely mainly on the shape, color, and/or texture features as well as their combinations, most of which are problem-specific and have shown to be complementary in medical images, which leads to a system that lacks the ability to make representations of high-level problem domain concepts and that has poor model generalization ability. Recent deep learning methods provide an effective way to construct an end-to-end model that can compute final classification labels with the raw pixels of medical images. However, due to the high resolution of the medical images and the small dataset size, deep learning models suffer from high computational costs and limitations in the model layers and channels. To solve these problems, in this paper, we propose a deep learning model that integrates Coding Network with Multilayer Perceptron (CNMP), which combines high-level features that are extracted from a deep convolutional neural network and some selected traditional features. The construction of the proposed model includes the following steps. First, we train a deep convolutional neural network as a coding network in a supervised manner, and the result is that it can code the raw pixels of medical images into feature vectors that represent high-level concepts for classification. Second, we extract a set of selected traditional features based on background knowledge of medical images. Finally, we design an efficient model that is based on neural networks to fuse the different feature groups obtained in the first and second step. We evaluate the proposed approach on two benchmark medical image datasets: HIS2828 and ISIC2017. We achieve an overall classification accuracy of 90.1% and 90.2%, respectively, which are higher than the current successful methods.

## 1. Introduction

With the rapid development of digital image acquisition and storage technologies, image understanding by computer programs has become an attractive and active topic in the machine learning field and in application-specific studies [1]. Toward building an intelligent Computer-Aided Diagnosis (CAD) system, fast and accurate annotation or the grading of medical images has become a key technique in CAD systems, in most medical fields. For example, many people are diagnosed with skin cancer in the United States every year [2]. If detected at an earlier stage, it would save many lives. A large number of research papers are reported in the area of medical image classification. However, medical images obtained from different sources may be variant from focusing region, contrast, and white balance. In addition, medical images usually have inner structures with different textures and pixels density. If we used only traditional features to classify medical images, it would be difficult to characterize certain classes efficiently [3]. In the past few years, deep learning has become one of the hottest research areas in computer science and computer applications. Because of the advances in deep learning, many researchers have attempted to use this new technique to address nonmedical images. Hinton et al. [4] first discussed the framework of the deep model. Henceforth, a variety of deep models have been proposed to solve image problems. Krizhevsky et al. [5] trained the deep convolutional neural network to classify images in ImageNet Large-Scale Visual Recognition Challenge 2010 (ILSVRC-2010), achieving state-of-the-art performance. In Reference [6], the authors discussed the effect of the deep mode depth on the performance in an image recognition task. Inspired by these successful studies, this approach has already attracted considerable work on leveraging this new methodology to solve medical image classification problems.

Medical image classification is one of the most important problems in the image recognition area, and its aim is to classify medical images into different categories to help doctors in disease diagnosis or further research. Overall, medical image classification can be divided into two steps. The first step is extracting effective features from the image. The second step is using the features to build models that classify the image dataset. In the past, doctors usually used their professional experience to extract features to classify the medical images into different classes, which is usually a difficult, boring, and time-consuming task. This approach is prone to leading to instability or nonrepeatable outcomes. Considering the research until now, medical image classification application research has had great merit. The researchers' efforts have led to a large number of published studies in this area. However, at present, we still cannot accomplish this mission efficiently. If we could finish the classification work excellently, then the results would help medical doctors to diagnose diseases with further study. Therefore, how to effectively solve this task is of great importance.

Considering past work, we have observed that a large number of previous studies [7–13] used shallow models for medical image classification, which rely mainly on the shape, color, and/or texture features as well as their combinations, before deep architectures appeared. However, for all of these models, the largest problem is that the extracted features are often referred to as low-level features; these features lack representation ability for high-level problem domain concepts, and their generalization ability is rather poor. In contrast, deep architectures [14–16] have achieved a large amount of success in the nonmedical image field. Deep learning-based methods, which are the most breathtaking branch of the machine learning field, provide an effective way to construct an end-to-end model that can compute final classification labels with the raw pixels of medical images. The applications of deep models in the medical image analysis domain require great effort to catch up with other areas of imaging because deep architectures require large datasets to obtain outstanding features. However, medical images are usually difficult to acquire, and thus, medical datasets are typically relatively small. Therefore, the approach is apt to lead to overfitting of the model if we directly use a deep model to address a small dataset. Except for these problems, the interpretability of the model has been proven to be rather poor, and training a deep model usually requires a large amount of computation. To overcome these concerns about traditional methods versus deep models, we present a special novel deep model that combines traditional features; this model can not only take full advantage of the existing doctors experiences but also utilize deep architectures to automatically extract high-level features for classify the medical images.

In this paper, we will focus on this novel and effective method of learning multiscale features that combine deep models with traditional image characteristics, which is referred to as CNMP. The main reason for applying this method to medical image classification is that we want to help doctors save time and energy by automatically classifying images. Moreover, it is noteworthy that the notable factor of our method is extracting features from the corresponding images, for which the deep model can automatically extract features and the traditional algorithms manually extract handcrafted features. This approach can simultaneously use both high- and low-level representations of an image and avoid any single representation or feature. Furthermore, it can automatically fuse two types of features, thus avoiding tiresome parameter selection.

There are at least two challenges to medical image classification, as follows:

- How can we extract effective features from a small medical image dataset? In general, medical image datasets are so small that we cannot obtain sufficient information to extract discriminative features. Without regard to the size of the image dataset, even if the proposed method can gain very good classification accuracy, the actual application value is extremely limited. In Reference [5], a new data augmentation method is proposed to avoid small datasets leading to acquiring nonvalid features. Then, they used an extension dataset to gain good performance of their model. Therefore, it is meaningful to find a method that can extract discriminative features from a small dataset.
- How to quickly and efficiently fuse different types of features from different models? It appears easy to formulate the idea of directly combining the feature vectors into a larger feature vector and determining one proportion parameter between different features. However, this method usually requires trial experiments to train the parameters and cannot obtain a better outcome. If we could design a more favorable fusion approach, then it would gain better accuracy performance than any of these methods. Therefore, there is a great demand to effectively fuse the features.

The main contributions of this paper can be summarized as follows:

- We have proposed a deep model that combines high-level features with traditional features to classify medical images. It directly trained the deep convolutional neural network called the coding network to extract high-level features rather than using domain-transferred convolutional neural networks such as domain-transferred convolutional neural networks (DT-CNNs) described in [17]. By means of adding traditional medical image features, the interpretability of the deep model and achieve the best performance could be improved.
- We have implemented two approaches to fusing the high-level features and traditional features. One method is to assign a fixed argument representation of the proposition between the high-level features and traditional features, in that the traditional procedure is boring, time-consuming, and difficult to put into practice. To conquer these limitations, another approach is proposed, which is a new framework that can not only fuse the features together but also automatically adjust their proportions.

The remainder of this paper is organized as follows: we review the previous related work on image classification in Section 2. The detailed description of the algorithm will be described in Section 3.  Section 4 presents the experimental results on two different datasets. Last, conclusions and future work are given in Section 5.

## 2. Related Work

There are many methods that have been proposed to solve these challenging problems on image classification, which can be categorized into two types of methods: traditional methods and deep model methods. Traditional methods include color and texture [7–10], random forests [11], and support vector machines [12, 13]. Studies on deep models to classify medical images include [14–16]. In this section, we will first give a detailed introduction to the previous work on image classification. Then, some literature [17–19] about feature fusion for image classification tasks will be reviewed.

In Reference [7], the authors have addressed two systems for the detection of melanomas in dermoscopy images using texture and color features. One system uses global features to classify skin lesions, and another system employs local features. The results were demonstrated on a dataset of 176 dermoscopy images from Hospital Pedro Hispano. Iyatomi et al. [8] proposed an Internet-based melanoma screening system based on shape, color, and texture features. This system gained a sensitivity (SE) of 86% and a specificity (SP) of 86% on 1200 dermoscopy images. Stoecker et al. [9] analyzed the areas of granularity between melanoma and similar areas in nonmelanoma skin lesions with a combination of color and texture features. Their paper used the receiver operating characteristic (ROC) curve to display the systems best separation performance on a dataset with 88 melanomas and 200 nonmelanoma lesions. Riaz et al. [10] first deployed a novel region-based texture and color descriptors to identify cancer in images. In their model, texture features are based on Gabor filters, and they use homomorphic filtering to obtain color features, which can address the problem of different rotations, scaling, and illumination.

Ramirez et al. [11] proposed a variant of random forests on single photon emission computed tomography (SPECT) image classification to help diagnose Alzheimer's disease (AD). First, they extracted score features using partial least squares from the image datasets to structure the random forests. Using this system as a classifier helped to classify all of the images. The specific process is to classify the image to the closest centroid recessively until reaching a leaf of a single tree, which is the classification of the image. The most important characteristic of this algorithm is that it can extend from the previous model, a process referred as to incremental learning, without retraining the images from scratch.

In Reference [12], the authors proposed a classifier that is based on a fractional Fourier transform and nonparallel support vector machine to classify magnetic resonance brain images into pathological brain image and healthy brain image categories. Thus, it was a binary classification task. For a given image, the system first used a weighted-type fractional Fourier transform to extract the spectrum features, and then, it utilized principal component analysis to reduce the dimensionality of the spectrum features. Finally, its incorporated spectrum features were fed into support vector machines. However, in this paper, the dataset that contains 90 T2-weighted MR brain images is relatively small. Although it has obtained good performance, it is clear that it is not adapted to a larger dataset.

Li et al. [14] trained a customized convolutional neural network (CNN) to classify lung image patches. In this model, the system contained only one convolutional layer to extract the deep features, to overcome the overfitting problem, and it obtained the best classification performance compared with scale-invariant feature transform (SIFT) features, rotation-invariant local binary pattern (LBP) features, and unsupervised feature learning using the restricted Boltzmann machine (RBM). In Reference [20], the authors first proposed simple deep learning architecture called principal component analysis network (PCANet) that had been used by [21] combined with the spatial distribution information of color images to achieve the state-of-the-art classification accuracy in various databases. In Reference [15], the authors employed a CNN trained by ImageNet to identify different types of pathologies in chest X-ray images. They achieved the best accuracy performance by combining the features extracted from a CNN and handcrafted features. Shin et al. [16] discussed why transfer learning can be useful to address medical images. Additionally, they proved their results on thoracoabdominal lymph node (LN) detection and interstitial lung disease (ILD) classification. Rakotomamonjy et al. [22] employed scattering transform which first proposed by [23] to extract features combined with local binary patterns (LBP) and local quinary patterns (LQP) for lung cancer detection which proved to be robust to small deformations in the images. And they verified the performances and effectiveness on the 2D-Hela dataset and Pap smear dataset. Cruzroa et al. [24] presented a deep learning approach for automatic detection of invasive ductal carcinoma (IDC) tissue regions in whole slide images (WSI) of breast cancer (BCa) which verified through a dataset from 162 patients diagnosed with IDC achieving balanced accuracy 84.23%.

Ahn et al. [17] proposed a method that combined domain-transferred convolutional neural networks (DT-CNNS) with a sparse spatial pyramid (SSP) to classify X-ray images. In this paper, they used VGG19 (19 layers CNN) proposed by [6] as the transferred network, which could ignore the medical image characteristics. However, this approach provided a new train of thought to solve this problem. In Reference [25], the authors first proposed multiscale high-level feature representations for face verification, which they termed Deep hidden Identity features (DeepID). The multiscale features fuse the features extracted from the third and fourth layers of the CNN model. In Reference [19], the authors presented a logistic regression-based fusion method that can fuse shape and color features without being tied to any of them. Their model implicitly weighted the visual words to overcome the shortcomings of not considering their statistical dependences. Li et al. [26] employed kernel principal component analysis (KPCA) as the fusion method to find nonlinear relationships of the extracted color and texture features which is then used as the maximum likelihood approach for automatic selection of optimal feature set from the fused feature.

All of the above algorithms have some problems in that they used transferred convolutional neural networks or directly employed traditional method to classify the medical images. In traditional methods, regardless of which features (color features, texture features, or shape features) are used, it is not adequate to classify the medical images solely by those features that are gained through experience. For deep models, the transfer-learning network finds it very easy to ignore the characteristics of medical images. In addition, most of the literature about medical image classification is on binary classification. In practice, we usually need to perform a multiclass classification task. To solve these problems and improve the performance of medical image classification, we present our new algorithm.

## 3. Methodology of the Proposed Model

**Figure 1:**
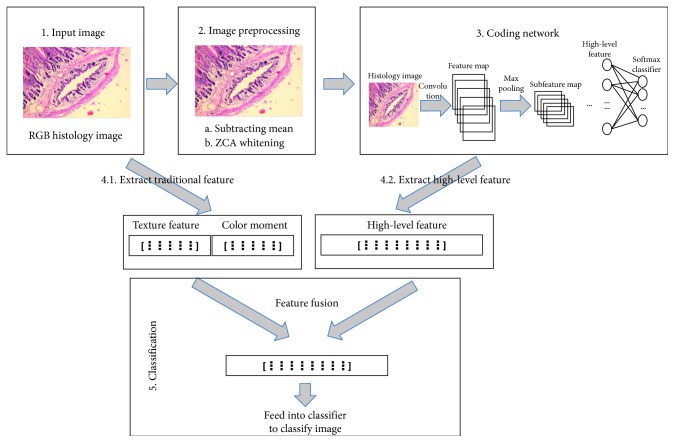
Framework of the approach.

In this section, we will highlight the key components of the CNMP model, providing a description of the coding network and the traditional feature extraction in subsections 3.1 and 3.2. In subsection 3.3, we introduce the detailed fusion process.  Figure 1 shows the detailed procedure of our algorithm.

Since the detailed introduction of LeNet-5 [27] in the 1990s, the convolutional neural network (CNN) has been widely used in image classification, video recognition, and object detection, and it has achieved the excellent performance in these areas. The CNN usually contains convolutional layers, pooling layers, one or more fully connected layers, and a softmax layer. The convolutional layers combined with the pooling layers are used for extracting features. The softmax layer is regarded as the classifier. The main design principles of the deep model are as follows: (1) to perform the image preprocessing, such as subtracting the mean RGB value and ZCA whitening; (2) choosing the proper activation function [28]; (3) the initial weights are also important. If the initial weights are too small, then the deep network would not be able to learn, and if they are too large, then the initial weights would undergo divergence [29]; (4) data augmentation, such as extracting random patches from the original image, horizontally flipping them in the image [5], which is especially important in medical image analysis; (5) using dropout to reduce overfitting and local response normalization [5] to reduce error rates are equally important; (6) choose the proper learning rate. To decay the learning rate in each epoch is the common usage; (7) deep network architecture is the most important principle. This would be confirmed on [30] and [6] that they achieved the state-of-the-art results on ILSVRC-2013 and ILSVRC-2014, respectively. According to the above patterns, we train a CNN as a coding network in the following way.

### 3.1. Coding Network

#### 3.1.1. The Structure of a Coding Network

When training the coding network, the input of the coding network is a fixed-size 140 × 140 RGB image. Before feeding the medical image into the network, every image pixel value subtracts the mean RGB value. The coding network consists of a series of convolutional layers and pooling layers. The convolutional layer employs a filter with a receptive field of 11 × 11, 9 × 9, and 8 × 8 and a 1 pixel stride and 0 pixel padding. The convolution operation is defined as follows:

(1) $$\begin{array}{c} y_{j}^{r}=f\left(b_{j}^{r}+\sum w_{i,j}^{r-1}\;*\;x_{i}^{r}\right). \end{array}$$

Here, *r* is the r-th layer in the coding network and *f* is the activation function, which we would discuss in the next subsection; *x*_*i*_ and *y*_*j*_ are the i-th input feature map and the j-th output feature map; *w*_*i*,*j*_ is the convolution kernel between *x*_*i*_ and *y*_*j*_; *b*_*j*_ is the bias; and *∗* is the convolution operation. The pooling layer is performed by max-pooling with a 5 × 5 window with a stride of 2 that signifies the overlapping pooling. The pooling operation is defined as

(2) $$\begin{array}{c} y_{j,k}^{i}=\underset{0\leq m,n<5}{\max}\left(x_{j\cdot 5+m,k\cdot 5+n}^{i}\right), \end{array}$$

where each element in the output feature map *y*^*i*^ is pooling from the 5 × 5 overlapping local region in the input feature map *x*^*i*^. At the last layer of the coding network, we use softmax as a classifier to classify the medical image.  Table 1 shows the detailed configuration of the coding network.

#### 3.1.2. Activation Function

The original activation functions are the sigmoid function *f*(*x*)=(1+*e*^−*x*^)^−1^ and tanh function *f*(*x*)=tanh(*x*); their derivatives can be expressed by themselves, and they can map the larger change values into a smaller range. However, the two functions have common problems in that the convergence rate is rather slow and there is a gradient diffusion problem. To make them computationally efficient and reduce the gradient diffusion effect, we will follow Hinton [28] to use Rectified Linear Units (ReLus) *f*(*x*)=max(0, *x*) as the activation function of the coding network. Additionally, in this paper, authors turn out that ReLus can converge faster than sigmoid or tanh.

#### 3.1.3. Softmax

At the last layer of our network, we connect in a softmax layer, which can predict *n* different classes through computing the probability of belonging to each category. In the last layer within our network, the feature will rasterize into *x*, which is a column feature vector:

(3) $$\begin{array}{c} p\left(y=j\left|x,\;\theta \right.\right)=\frac{e^{\theta_{j}^{T}x}}{{\sum^{}}_{j=1}^{k}e^{\theta_{j}^{T}x}}, \end{array}$$

where the target contains *k* classes, and *θ*_*j*_^*T*^ is the weight vector.

### 3.2. Traditional Feature Extraction

According to Reference [7, 8, 31, 32], the most commonly used features in medical image classification include shape features, color histogram features, color moment features, and texture features. Most of the previous studies employ global features to classify medical images. The texture and color moment are the most commonly used features that are used in identifying targets, and they are together global features. Celebi et al. [32] extracted color, texture features, and added shape features that were fed into an SVM classifier, achieving SE = 93% and SP = 92%. Rubegni et al. [33] used color moment features and texture features to achieve good performance on a dataset with 217 melanomas and 588 images. Therefore, we follow the authors to employ the color moment and texture features as the traditional features. Texture is a statistical distribution feature that can describe the innate properties of an image surface. It is based on multiple pixel area computing instead of single pixels. Instead, the color moment is based on a single pixel. It is not very sensitive to the angle or size of the image.  Table 2 lists the commonly used terminologies in this section.

To compute the image texture features, we will first acquire the gray-level co-occurrence matrix **G** by the image itself, which can compute the statistics of pairs of neighboring pixels [34]. Then, we employ the angular second moment (ASM), entropy (ENT), contrast (CON), and correlation (COR) to signify the texture features that can be derived by the matrix **G**, which fix the distance between two pixels to one with the angle at 0°, 45°, 90°, or 135°. The definitions are as follows:

(4) $$\begin{array}{c} \mathrm{ASM}=\overset{s}{\underset{i}{\sum }}\overset{s}{\underset{j}{\sum }}G{\left(i,j\right)}^{2}. \end{array}$$

The angular second moment is the sum of squares of every element in the matrix **G**. It is representative of homogeneity of the image and roughness of the texture. If the elements in matrix **G** are almost the same, then the ASM value will be small. Otherwise, the ASM will be large.

(5) $$\begin{array}{c} \mathrm{ENT}=-\underset{i}{\sum }\underset{j}{\sum }G\left(i,j\right)\;\log\left(G\left(i,j\right)\right). \end{array}$$

Entropy is a measurement of the uncertainty, and it can be used to denote the uncertain information about the image. When all of the elements in matrix **G** are equal, the image contains the largest amount of uncertain information based on the largest ENT value. By this time, the distribution of the gray values in the image is very complicated.

(6) $$\begin{array}{c} \mathrm{CON}=\underset{i}{\sum }\underset{j}{\sum }{\left(i-j\right)}^{2}G\left(i,j\right). \end{array}$$

Contrast is a measure of how the data in the image are distributed or the image clarity. The larger the value of CON, the clearer the image.

(7) $$\begin{array}{c} \text{COR}=\frac{{\sum^{}}_{i}^{s}{\sum^{}}_{j}^{s}ijG\left(i,j\right)-\mu_{x}\mu_{y}}{\sigma_{x}\sigma_{y}}. \end{array}$$

After calculating ASM, ENT, CON, and COR, we continue to determine the mean and standard deviation of each of the others, which results in a texture feature vector. The feature vector will be used together with the color moment as the traditional features.

Color moment features are always represented by the mean, standard deviation, and the third-order color moment. The mean will display the lightness or darkness of the image; the standard deviation can reflect the range of the image color distribution; and the third-order color moment shows the symmetry of the image color distribution. Therefore, this approach leads to a color moment feature vector. The color moment definition is as follows:

(8) $$\begin{array}{c} A_{i}=\frac{1}{N}\overset{N}{\underset{j}{\sum }}P\left(i,j\right),\\ V_{i}=\sqrt{\frac{1}{N}\overset{N}{\underset{j}{\sum }}{\left(P\left(i,j\right)-A_{i}\right)}^{2}},\\ S_{i}=\sqrt[{3}]{\frac{1}{N}\overset{N}{\underset{j}{\sum }}{\left(P\left(i,j\right)-A_{i}\right)}^{3}}. \end{array}$$

### 3.3. Feature Fusion

After extracting the high-level features and traditional features, we design two different fusion approaches to fuse the features. The first method is to set a fixed proportion λ, which calls the *R* feature fusion. The fusion feature for classification is computed as follows:

(9) $$\begin{array}{c} \mathrm{NF}=\lambda \cdot \mathrm{LF}+\left(1-\lambda \right)\cdot \mathrm{HF}, \end{array}$$

where NF is the fusion feature, and LF and HF indicate traditional features and high-level features, respectively. The *λ* is the weight parameter that signifies the importance between two different features. This method is very easy to implement because it is locally weighted. Once we have obtained the parameter *λ*, there is no need to recalculate. The feature fusion will feed into softmax to accomplish the last classification task. However, this method is only for linear feature fusion, and it required a large number of experiments to obtain the parameter *λ*. Above all, it is difficult to fuse the features to effectively represent the images. Additionally, if it changes to another dataset, then the same experiment is required to obtain the parameter *λ* again.

To solve these problems, we propose another approach, which can automatically adjust the proportion between high-level features and traditional features, to avoid the boring, time-consuming process of obtaining the parameter *λ*. The method is to train a multilayer perceptron neural network that can fuse the features in nonlinear feature space. The fusion feature (RF) operation is defined as follows:

(10) $$\begin{array}{c} \text{RF}=\max\left(0,\;\;\overset{n}{\underset{i}{\sum }}w_{i}l_{i}+\overset{m}{\underset{j}{\sum }}w_{j}h_{j}+b\right), \end{array}$$

where LF={*l*_1_, *l*_2_, …, *l*_*i*_, …, *l*_*n*_} and HF={*h*_1_, *h*_2_, …, *h*_*j*_, …, *h*_*m*_} represent traditional and high-level features and *b* is the bias. The multilayer perceptron contains a fully connected layer and a softmax layer as a classifier. It is consistent with the kernel function idea, which would map low-dimensional information into high-dimensional information. Therefore, it can gain better discriminative features for medical images than using a linear feature space. This approach will be further demonstrated in the following experiments. In addition, it may greatly reduce the amount of computation in that it would not attempt to determine the same parameter again and again.

## 4. Experiment and Evaluation

We implemented the coding network to extract the high-level features in MatConvnet, which is a matlab toolbox that implements convolutional neural networks, as well as extracted the traditional features based on color moment and texture. We have designed a series of experiments to verify the effectiveness of our method on two benchmark medical image datasets. One is the HIS2828 dataset, and the other is the ISIC2017 dataset. We conducted all of our experiments on a computer with i5-6500 3.2 GHz CPU, 32G main memory, and GTX1060 GPU.

### 4.1. The HIS2828 and ISIC2017 Datasets

The HIS2828 dataset is composed of 4 classes of fundamental tissue images that are representative of different tissue types. Each image is an RGB image of size 720 ∗ 480. This dataset contains 2828 images, which can be listed as follows: 1026 nervous tissue images, 484 connective tissue images, 804 epithelial tissue images, and 514 muscular tissue images, in which we utilize 1, 2, 3, and 4 to represent the labels.  Table 3 displays the composition of the HIS2828 dataset.

ISIC2017 is a dataset of skin lesions that is provided by The International Skin Imaging Collaboration(ISIC). It includes 2000 images; 374 of them are malignant skin tumors referred to as ”Melanoma” and 1626 of them are benign skin tumors referred to as ”Nevus of Seborrheic Keratosis”. Thus, it is a binary image classification task that distinguishes between (a) Melanoma and (b) Nevus and Seborrheic Keratosis. Each image in this dataset has a different resolution, which we must address.  Table 4 shows the composition of the ISIC2017 dataset.

In order to evaluate our experiments, we employed the following configuration. First, each dataset was divided into a training set, a validation set, and a test set with the ratio 7 : 1 : 2. Then, all the methods were evaluated using 10-fold cross validation. After that, the images were randomly cropped from the original dataset in order to obtain fixed-size 140 × 140 images for feeding them into the coding network. For HIS2828 dataset, each image was randomly cropped to 420 × 420 and then resized to the fixed-size 140 × 140 image. However, for ISIC2017 dataset, prior to resizing to 140 × 140, we extracted random patches with two-thirds of the original height and width for images having different resolutions. This would save the image information to a great extent and reduce the computation complexity effectively. These works can not only obtain the fixed-size but also augment the image samples. In addition, we would flip the image horizontality or verticality to further amplify the image datasets. At test time, the network makes a prediction to each patche and averaging of the predictions made by the softmax layer if the patches belongs to the same image. The influence of image augmentation on the accuracy and running time will be discussed in the following experiments.

The network architecture of our coding framework is presented detail in Table 1. As shown in the Table 1, it is able to converge after 45 epoches. Finally, for each convolutional layer, we used ReLus as an activation function. Besides, batch normalization was also employed in order to accelerate deep network training.

### 4.2. Accuracy

In this section, we will provide a series of experiments on the accuracy and algorithm running time on two real medical image datasets. The accuracy here is defined as the percentage of correctly classified medical images. To better compare the algorithms, we employ the confusion matrix and receiver operating characteristic (ROC) curve to further evaluate the model. The confusion matrix is a table layout that can describe the number of true positive, false negative, true negative, and false negative in an evaluation of a multiclass image classification algorithm. The ROC curve is a graphic plot that is obtained through computing the true positive rate (TPR) against the false positive rate (FPR) by setting different thresholds, where the definition of TPR and FPR are as follows:

(11) $$\begin{array}{c} \text{TPR}=\frac{\text{TP}}{\text{TP}+\text{FN}},\\ \text{FPR}=\frac{\text{FP}}{\text{FP}+\text{TN}}, \end{array}$$

where TP, FN, FP, and TN are true positive, false negative, false positive, and true negative, respectively. It is very useful to exhibit the performance of the binary class image classification algorithm. In the following, the support vector machine (SVM) is determined to be a universal classifier in machine learning before the appearance of the deep learning model; therefore, the SVM (traditional feature) and SVM (traditional and deep feature) that is the concatenation of traditional and deep feature will be compared with the CNMP model. Here, we employ the package of LibSVM-3.17 to train a one-vs-one multiclass classifier with radial basis function (RBF) kernal. To demonstrate the effectiveness of combining features, a comparison with the coding network becomes necessary. Furthermore, compared with *R* feature fusion and KPCA feature fusion will show that the CNMP contains a better feature fusion approach. The reason why we choose KPCA with the RBF kernal to fuse features is that it could map the feature into nonlinear space as well. The feature fusion vector will feed into softmax to finish classification task.

The results on the HIS2828 and ISIC2017 datasets with regard to the accuracy are shown in Table 5. Our method achieves the best accuracy rate, which is 90.2% and 90.1%, respectively, on the two datasets. From the table, we can see that the accuracy of SVM (traditional feature) is the lowest. Even if we utilize the coding network to classify the medical image, it would achieve better performance. This finding proves that high-level features can represent a medical image better than traditional features. Our model obtains higher accuracy than the two previous methods, and thus, combining the two different types of features can work very well because the combined features would signify the images from a multiscale perspective. It is obvious that SVM (traditional and deep feature) may work better than coding network and SVM (traditional features). In addition, comparing our model with the *R* feature fusion and KPCA feature fusion, we can conclude that automatic feature fusion not only obtains better performance but also avoids the tedious process of manually adjusting the parameters.

**Figure 2:**
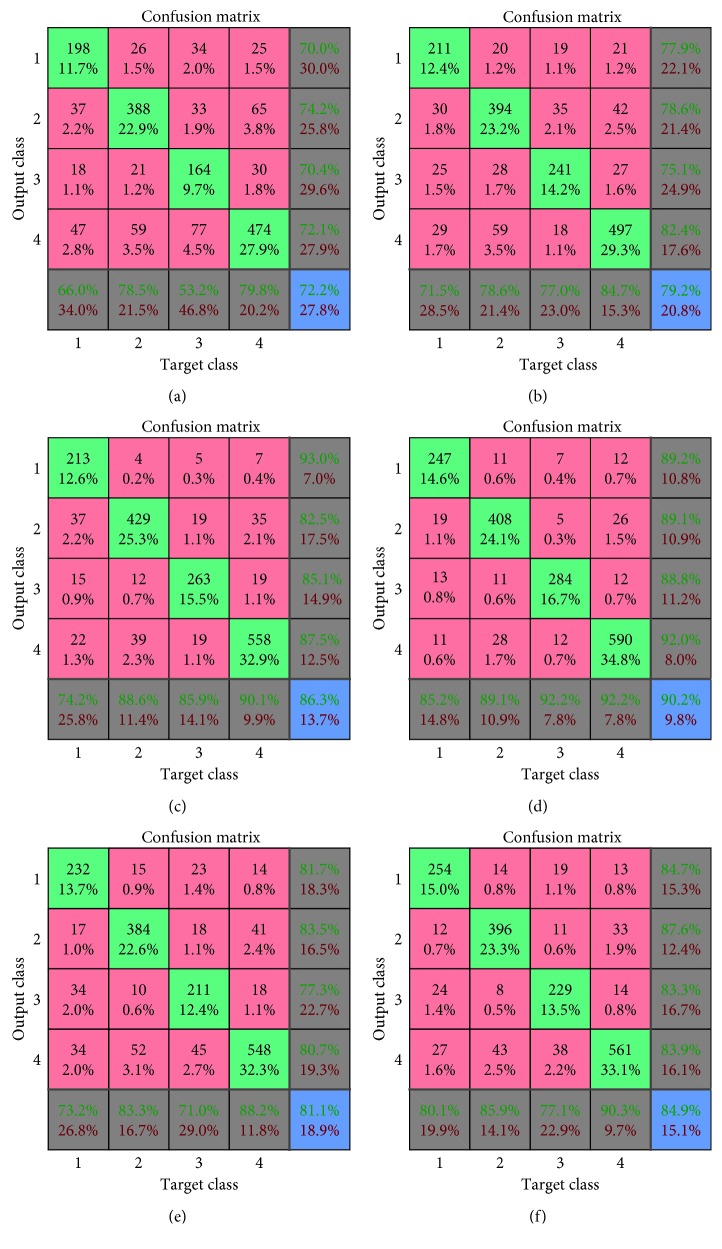
Comparison of the confusion matrix on the histology dataset. (a) The confusion matrix of SVM (traditional features). (b) The confusion matrix of coding network. (c) The confusion matrix of R feature fusion. (d) The confusion matrix of CNMP. (e) The confusion matrix of SVM (traditional and deep feature). (f) The confusion matrix of KPCA feature fusion.

As is known, the overall accuracy cannot be an appropriate measure for evaluating an image classification algorithm, especially when the image dataset has the problem of having an imbalanced distribution. It is obvious that the HIS2018 dataset has the sample imbalance problem. Here, with the purpose of making a better comparison, we employ the confusion matrix to further evaluate the algorithms. In confusion matrix, the first four diagonal cells represent the number and percentage of correct predictions made by the model on the test set. The pink-shaded cells illustrate incorrect predictions, and the percentage corresponds to all data in test set. The gray-shaded cells in the last column of the matrix show the percentage of correctly identified positive predictions called the sensitivity or recall rate, while the gray shaded cells in the last row represents the precision rate of each class. Finally, the last diagonal cell represents the overall accuracy.  Figure 2 demonstrates that since nervous tissue and epithelial tissue have more training samples, they can gain higher precision and recall. Moreover, from Figure 2(d), the CNMP algorithm has the highest precision and recall in every category, which proves the efficiency of our model. In Figure 2(c), *R* feature fusion has the second highest performance, which is close to CNMP in nervous tissue and epithelial tissue, with the exception of the other two categories; Figure 2(a) and Figure 2(b) show that the coding network can obtain a better outcome than SVM (traditional features). However, the unemployed multistage features restrict the SVM (traditional features) and coding network from achieving better performance. In addition, Figure 2(a) states that the SVM (traditional features) is the most easily affected by the imbalance problem. From Figure 2(c) and Figure 2(e), if it directly concatenated the features, it is a really bad practice. Comparing Figure 2(f) with Figure 2(d) proves the effectiveness of our fusion strategy again. In general, when the image dataset has an imbalance problem, it is possible for the SVM (traditional features) to obtain poor generalization ability. Instead, the deep model would be good at avoiding this problem to obtain a better outcome.

**Figure 3:**
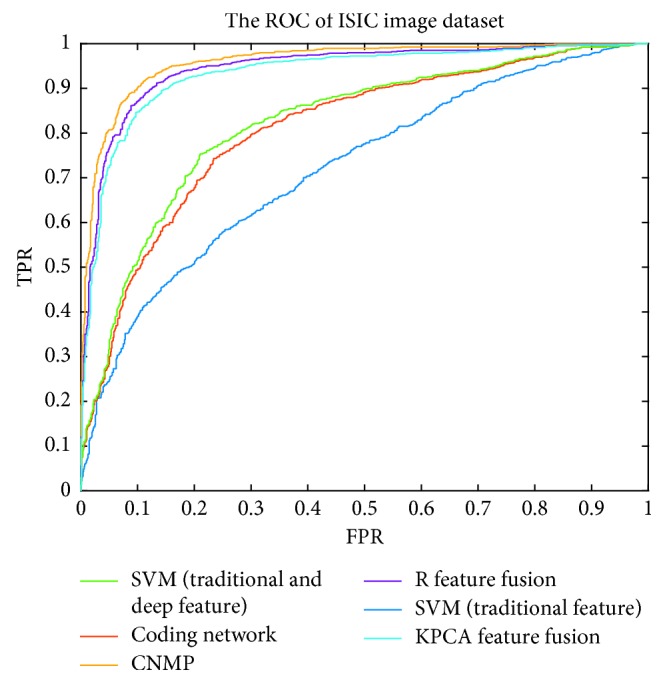
The ROC curve on the ISIC2017 dataset.

The receiver operating characteristic (ROC) curve is a graphic plot that is obtained through computing the TPR and FPR by setting different thresholds. It is useful to evaluate the performance of the binary class image classification algorithm since the binary dataset contains a sample imbalanced problem.  Figure 3 compares the ROC curve of different algorithms on the ISIC2017 dataset. The closer the curve is to the upper left corner of the axes, the better the performance. It is easy to see that our model obtains the best performance and that *R* feature fusion has the second best performance. To make a more intuitive comparison, we will determine the area under the curve (AUC) to signify the predictive performance; the larger the value of the AUC, the better the performance. Huang and Ling [35] have proven that the AUC is a statistically consistent and more discriminatory measure than the accuracy.  Table 6 shows the AUC values of the different algorithms. The AUC value of our model is the larger than that of the other three algorithms.

### 4.3. Running Time

**Figure 4:**
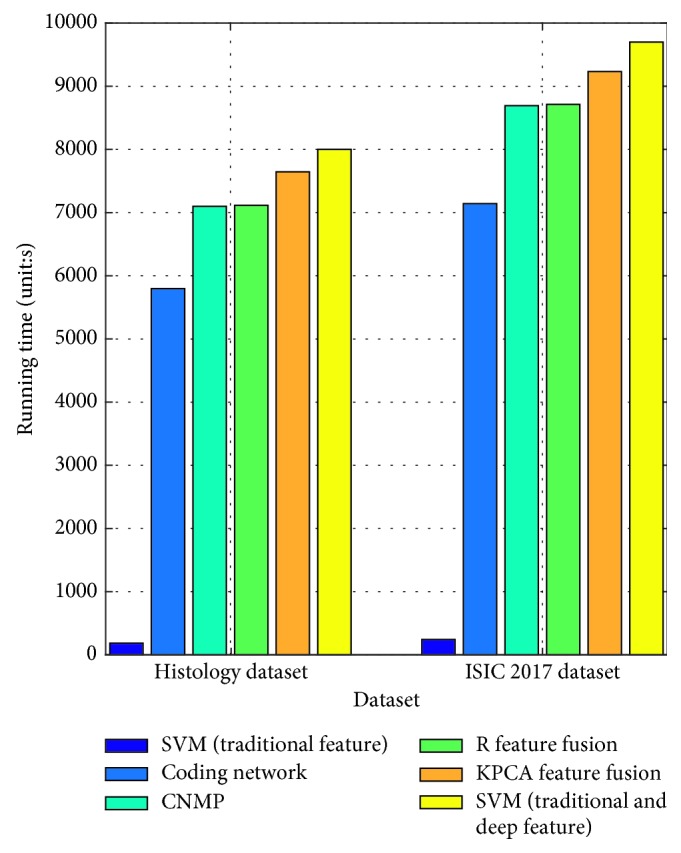
The running time of different algorithms.

In this section, we also exhibit the running time of the four algorithms. The comparison can be seen in Figure 4. In the figure, there is no surprise that the SVM (traditional features) can run the fastest. It has several factors that contribute to this accomplishment. (1) It uses little image information along with abandoning a large amount of the image's spatial information. (2) This model must train very few parameters with respect to the deep model. The coding network is the second fastest algorithm. However, its running time greatly exceeds that of SVM (traditional features). The reason for this finding is that the deep model must train a mass of parameters to improve the generalization ability of the model. In addition, it takes advantage of almost all of the information in the image, by integrating the features layer by layer. Furthermore, we can know from this figure that although the *R* feature fusion and CNMP can achieve the best accuracy performance, their running time is rather long. SVM (traditional and deep feature) has the longest running time because it needs great time to get the high-level feature and traditional feature. Besides this, it must overcome high dimensionality to obtain the classification model. For KPCA feature fusion, it takes great time to fuse the different features.

### 4.4. The Variation of *m*

**Figure 5:**
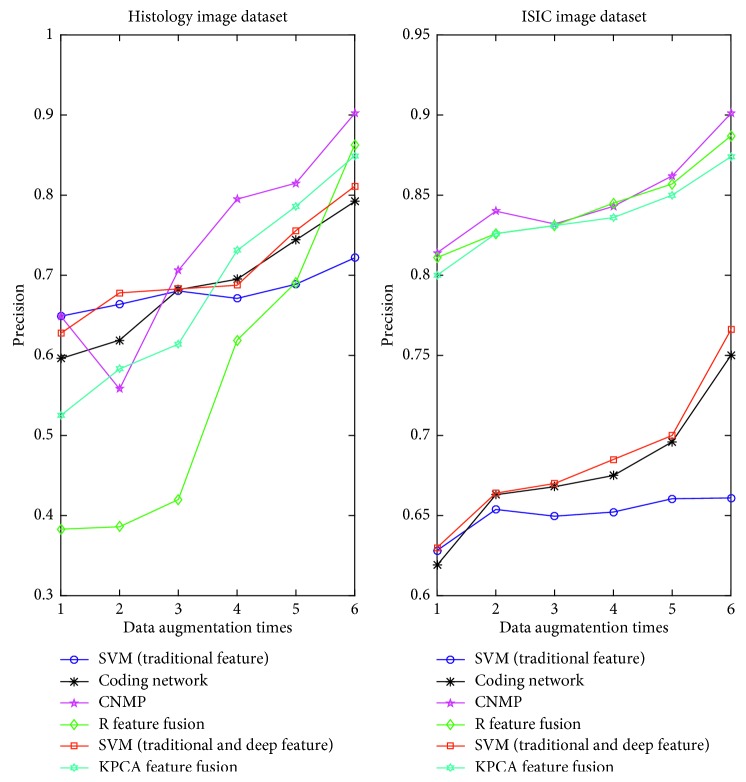
The variation *m* influence on algorithm's accuracy.

Here, we will conduct further study of how to expand the number of images that influence the algorithm's accuracy and running time. In reference [5], the authors determined that increasing the image samples would affect the deep model performance. Nevertheless, the authors did not further research this matter with qualitative analysis. The relationship between the image augmentation times (*m*) and the algorithm's accuracy are shown in Figure 5. We can clearly determine that when the medical image dataset is relatively small, there is no difference between the deep model and SVM (traditional features). Additionally, it is possible that SVM (traditional features) has better accuracy performance. As the number of images is gradually increased, deep models have been shown to have a powerful generalization ability, especially with our model gaining the best performance. However, from Figure 5, it appears strange that SVM (traditional features) is hardly influenced by the increasing number of images. Even the coding network obtained better accuracy than the SVM (traditional feature). Extracting traditional features that cannot be a good abstraction of medical images could explain this finding.

**Figure 6:**
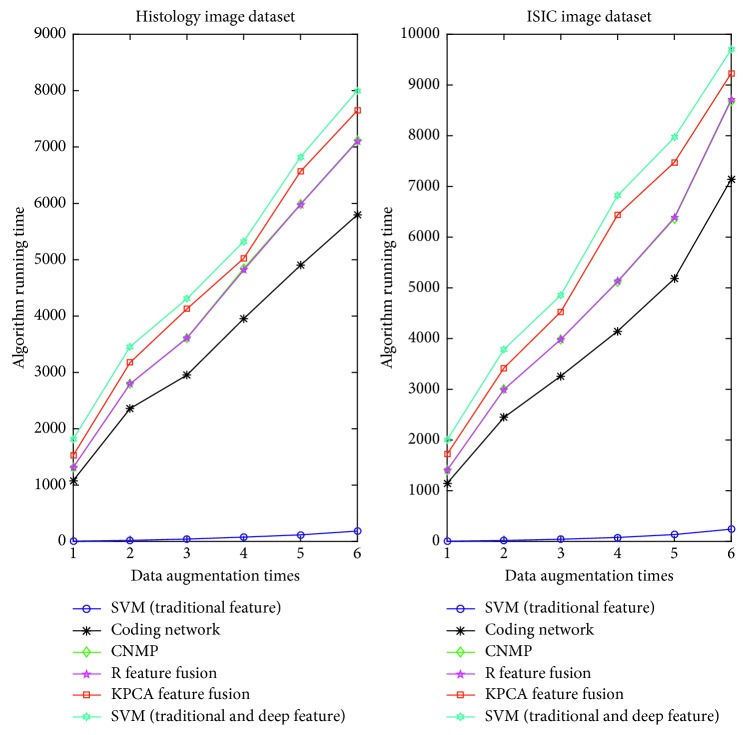
The variation *m* influence on running time.

Figure 6 shows that as the number of images increases, the running time of all of the algorithms increases. It is obvious that the deep model is not on the same order of magnitude as the SVM (traditional features). The running time of SVM (traditional features) is a linear correlation with the image numbers in a restricted range of time. However, although the running time of the deep model also has a linear relationship with the number of images, the line's slope is rather large. In addition, our model and *R* feature fusion have almost the same routine on the running time as the number of images increases. This finding proves our model's efficiency from the viewpoint of the running time.

## 5. Conclusions and Summary

In this paper, we presented a new medical image classification algorithm that combines high-level feature extraction from a coding network with traditional image features, and we call it CNMP. As far as we know, this study is the first time that a deep model has been directly utilized by including traditional image features to classify medical images. The experimental results show that our method can achieve an accuracy of 90.2% and 90.1% on the HIS2828 and ISIC2017 image datasets, which outperforms SVM (traditional features), coding network, and *R* feature fusion by considerable margins. Moreover, we discuss the influence of image extension on the algorithm's accuracy and running time. Future work could consider [36, 37] for adding an efficient pruning strategy to greatly reduce the parameters. In addition, we could employ the ”Network in Network”(NIN) [38] in the future, to gain better nonlinear high-level features for representations of medical images, which may achieve better performance than our model. In the aspect of feature fusion strategies, we are interested in developing more methods like multifeature fusion deep networks (MFFDN) [39], based on denoising autoencoder, or metaspace fusion to combine homogeneous representations [40].

## Figures and Tables

**Table 1:** The configuration of the coding network.

Type	Patch size/stride	Output size
Convolution	11 × 11/1	130 × 130 × 32
Convolution	11 × 11/1	120 × 120 × 32
Max pool	5 × 5/2	58 × 58 × 32
Convolution	9 × 9/1	50 × 50 × 64
Max pool	5 × 5/2	23 × 23 × 64
Convolution	8 × 8/1	16 × 16 × 128
Convolution	9 × 9/1	8 × 8 × 256
Convolution	8 × 8/1	1 × 1 × 256
Rasterize		1 × 1 × 4
Softmax layer		1 × 1 × 4

**Table 2:** Summary of the symbols.

Symbols	The detail description
*G*	The gray-level co-occurrence matrix
*s*	The size of *G*
*G*(*i*, *j*)	The *i*-th row *j*-th column element in *G*
*μ*_*x*_, *μ*_*y*_	*μ*_*x*_, *μ*_*y*_ are the means the marginal distribution of *G*
*σ*_*x*_, *σ*_*y*_	*σ*_*x*_, *σ*_*y*_ are the standard deviations of the marginal distribution of *G*
*P*	A matrix for representation of the image
*N*	The number of pixels in *P*
*P*(*i*, *j*)	The *j*-th pixel of the *i*-th channel in *P*
*A*_*i*_	The mean of the *i*-th channel in *P*
*V*_*i*_	The variance of the *i*-th channel in *P*
*S*_*i*_	The skewness of the *i*-th channel in *P*

**Table 3:** The composition of the HIS2828 dataset.

Image category	Number of images	Label
Nervous tissue	1026	1
Connective tissue	484	2
Epithelial tissue	804	3
Muscular tissue	514	4

**Table 4:** The composition of the ISIC2017 dataset.

Image category	Number of images	Label
Melanoma	374	1
Nevus of seborrheic keratosis	1626	2

**Table 5:** Comparison of the classification algorithms accuracy.

Algorithm	HIS2828	ISIC2017
SVM (traditional feature)	72.17%	66.1%
Coding network	79.5%	75%
CNMP	90.2%	90.1%
R feature fusion	86.3%	88.7%
SVM (traditional and deep feature)	81.1%	77.6%
KPCA feature fusion	84.9%	87.4%

**Table 6:** Comparison of the AUCs on the ISIC dataset.

Algorithm	The AUC of ROC
SVM (traditional feature)	0.7209
Coding network	0.8087
CNMP	0.9585
R feature fusion	0.9436
SVM (traditional and deep feature)	0.8210
KPCA feature fusion	0.9326

## Data Availability

The ISIC dataset is taken from the website https://challenge.kitware.com/. And the histology 2828 dataset is taken from http://www.informed.unal.edu.co/.
